# Capabilities of ChatGPT-3.5 as a Urological Triage System

**DOI:** 10.1016/j.euros.2024.10.015

**Published:** 2024-11-01

**Authors:** Christopher Hirtsiefer, Tim Nestler, Johanna Eckrich, Henrieke Beverungen, Carolin Siech, Cem Aksoy, Marianne Leitsmann, Martin Baunacke, Annemarie Uhlig

**Affiliations:** a Klinik und Poliklinik für Urologie, Universitätsklinikum Carl Gustav Carus Dresden, Dresden, Germany; b Klinik für Urologie, Bundeswehrzentralrankenhaus Koblenz, Koblenz, Germany; c Klinik und Poliklinik für Urologie und Kinderurologie, Universitätsklinikum Bonn, Germany; d St. Elisabeth Krankenhaus Leipzig, Leipzig, Germany; e Goethe University Frankfurt, University Hospital, Frankfurt am Main, Germany; f Klinik für Urologie, Universitätsklinikum Gießen und Marburg, Marburg, Germany; g Universitätsklinik für Urologie, Medizinische Universität Graz, Graz, Austria; h aQua-Institut für angewandte Qualitätsförderung und Forschung im Gesundheitswesen GmbH, Göttingen, Germany; i Klinik für Urologie, Universitätsmedizin Göttingen, Göttingen, Germany

**Keywords:** ChatGPT, Artificial intelligence, Triage, Urological emergency, Internet use

## Abstract

**Background and objective:**

Patients struggle to classify symptoms, which hinders timely medical presentation. With 35–75% of patients seeking information online before consulting a health care professional, generative language–based artificial intelligence (AI), exemplified by ChatGPT-3.5 (GPT-3.5) from OpenAI, has emerged as an important source. The aim of our study was to evaluate the role of GPT-3.5 in triaging acute urological conditions to address a gap in current research.

**Methods:**

We assessed GPT-3.5 performance in providing urological differential diagnoses (DD) and recommending a course of action (CoA). Six acute urological pathologies were identified for evaluation. Lay descriptions, sourced from patient forums, formed the basis for 472 queries that were independently entered by nine urologists. We evaluated the output in terms of compliance with the European Association of Urology (EAU) guidelines, the quality of the patient information using the validated DISCERN questionnaire, and a linguistic analysis.

**Key findings and limitations:**

The median GPT-3.5 ratings were 4/5 for DD and CoA, and 3/5 for overall information quality. English outputs received higher median ratings than German outputs for DD (4.27 vs 3.95; *p* < 0.001) and CoA (4.25 vs 4.05; *p* < 0.005). There was no difference in performance between urgent and non-urgent cases. Analysis of the information quality revealed notable underperformance for source indication, risk assessment, and influence on quality of life.

**Conclusion and clinical implications:**

Our results highlights the potential of GPT-3.5 as a triage system for offering individualized, empathetic advice mostly aligned with the EAU guidelines, outscoring other online information. Relevant shortcomings in terms of information quality, especially for risk assessment, need to be addressed to enhance the reliability. Broader transparency and quality improvements are needed before integration into, primarily English-speaking, patient care.

**Patient summary:**

We looked at the performance of ChatGPT-3.5 for patients seeking urology advice. We entered more than 400 German and English inputs and assessed the possible diagnoses suggested by this artificial intelligence tool. ChatGPT-3.5 scored well in providing a complete list of possible diagnoses and recommending a course of action mostly in line with current guidelines. The quality of the information was good overall, but missing and unclear sources for the information can be a problem.

## Take Home Message

GPT-3.5 can serve as a potential triage system for urological conditions. It offers good technical performance and individualized, empathetic advice mainly aligned with the European Association of Urology guidelines. However, broader research is warranted and quality improvements are needed before GPT-3.5 integration into patient care.

## 1 Introduction

Patients often struggle to identify and classify symptoms of acute illnesses and to rate the urgency of the need to seek a consultation. These difficulties might arise from limited professional insight as well as subjective concern. Depending on socioeconomic status, 35–75% of patients seek information on the internet about causes and consequences of their symptoms before consulting a health care professional [1].

Over the past decade, generative language–based artificial intelligence (AI) has emerged, which can serve as an additional source of information for patients. One widely known model is the OpenAI ChatGPT platform (https://chat.openai.com/). Recent studies have shown that 70% of individuals are willing to use ChatGPT for self-diagnosis and have high expectations for the quality of the information [2].

For common urological illnesses treated in the acute care setting [3], patients face a broad urgency spectrum, ranging from potentially life-threatening urosepsis caused by urolithiasis, to irreversible erectile dysfunction after untreated priapism, to barely any damage caused by prolonging presentation with a mild UTI. Some studies have evaluated the ability of GPT-3.5 to answer general health [4] or hypothetical patient-like questions [5] or to reflect guideline knowledge [6]. In a study that used text inputs based on real-world frequently asked questions on urolithiasis, 95% of the answers were considered satisfactory by two reviewing urologists [7]. Davis et al [8] created 18 hypothetical patient-like urological questions and found that 78% of the responses were appropriate, with a good overall score for text clarity.

All of these previous studies posed questions in various categories and had a low number of inputs with few repetitions, and only one systematically evaluated readability and clarity. Therefore, the aim of our study was to systematically evaluate the capabilities of GPT-3.5 as a triage system for patients with acute urological conditions before consulting a health care professional.

## 2 Materials and methods

The study had two primary endpoints: (1) the completeness and accuracy of urological differential diagnoses (DD) given realistic urological symptoms; and (2) the accuracy of the recommended course of action considering the input. As a secondary endpoint, we assessed the quality of the information generated by GPT-3.5 using the DISCERN questionnaire (http://www.discern.org.uk) [9], a well-established validated 16-item instrument that assesses the reliability, transparency, balance, and informativeness of publications on consumer health information.

From among the ten most commonly treated urological diseases [3], we identified three acute urological pathologies (Table 1) suitable for limited DD and triage decisions ranging from highly acute to treatable in primary care. We added testicular pain and masses and priapism because of the potentially serious consequences of delayed treatment. Pathologies were categorized as urgent (testicular pain, flank pain, priapism) or non-urgent (testicular mass, hematuria, urinary tract infection) to differentiate time-sensitive cases from those that usually do not need an immediate intervention.

**Table 1:** List of six common urological pathologies examined in the study and associated symptoms that were used to find corresponding user entries in online forums

Pathology	Associated symptoms
Testicular pain	Sudden onset of testicular pain, slow onset of testicular pain, fever, previous dysuria
Testicular mass	Painless testicular lump
Hematuria	Painful or painless macrohematuria, changed urine color
Urinary tract infection	Dysuria, alguria, pollakisuria, lower abdominal pain, flank pain, fever, chills, shivering
Flank pain	Colicky, severe flank pain, fever, groin pain
Priapism	Erection lasting more than 3 h

Online patient forums (Supplementary material) were searched for four or five real lay descriptions of each of the above six syndromes in German and in English (only 4 German descriptions were found for scrotal pain), resulting in 59 different input texts.

For assessment of the urological DD, the sentence “What could this be? Please name all the possible diagnoses!” was added to each description and entered into the GPT-3.5 input box.

Eight independent experienced urologists input all the queries on different servers located in Germany between July and October 2023. The input times varied, but were generally in daytime hours. Each lay description was entered as a separate query to avoid cross-reference to the previous input.

To assess the accuracy of recommendations on the course of action (CoA), the GPT-3.5 reply to the initial query was countered with “Given the previous information, would you suggest presenting in the Emergency Department immediately, at the GP [general practioner’s] office tomorrow or at the office of a urology consultant within the next week?”

The team of experienced urologists assessed compliance with the corresponding German guideline using a 5-item Likert scale (1 = not at all; 2 = in part; 3 = at least half; 4 = mostly; 5 = completely) for the DD and the CoA recommended by GPT-3.5.

To assess the competency of ChatGPT in providing accurate answers to the queries posed, we used questions 1 and 2 from Section 1 of the DISCERN instrument. Specifically, question 2 helps in determining whether the information about treatment choices promised by a publication at the start of the process was actually delivered. We modified the evaluation to determine whether the ChatGPT answer accurately addressed the query posed. We also assessed the outputs according to the 16 items of the DISCERN questionnaire [9], with items 1–8 applied for DD, items 9–15 for the recommended CoA, and item 16 (D16) for the overall quality of the output.

The procedure was performed once in German and once in English. Eight urologists (M.B., C.H., T.N., J.E., H.B., C.S., A.U., C.A.) entered and assessed the German queries. For linguistic reasons, the English outputs for queries entered by authors J.E. and C.S. were evaluated by authors M.B. and C.H. In total, 232 German and 240 English queries each consisting of two questions were entered and rated by nine urologists.

Statistical analysis included descriptive statistics, correlation tests, and assessment of inter-rater reliability (IRR). Data for continuous variables did not follow a normal distribution and were therefore compared using two-sided unrelated Mann-Whitney U tests. Correlation between output length and the rating score was assessed via nonparametric Spearman correlation. IRR for the ratings was evaluated using the Fleiss κ metric. As the team of researchers rating the outputs was rearranged for linguistic reasons, only six raters were included in the IRR analysis. If relevant, missing values were excluded by analysis, resulting in varying numbers of cases analyzed. The α level was set at 0.05. Statistical analyses were performed using SPSS v26 (IBM Corp., Armonk, NY, USA).

Ethics approval was not required as all the data are publicly available and were processed anonymously. The study conformed with all relevant aspects of the Declaration of Helsinki.

## 3 Results

### 3.1 Rating scores

**Fig. 1:**
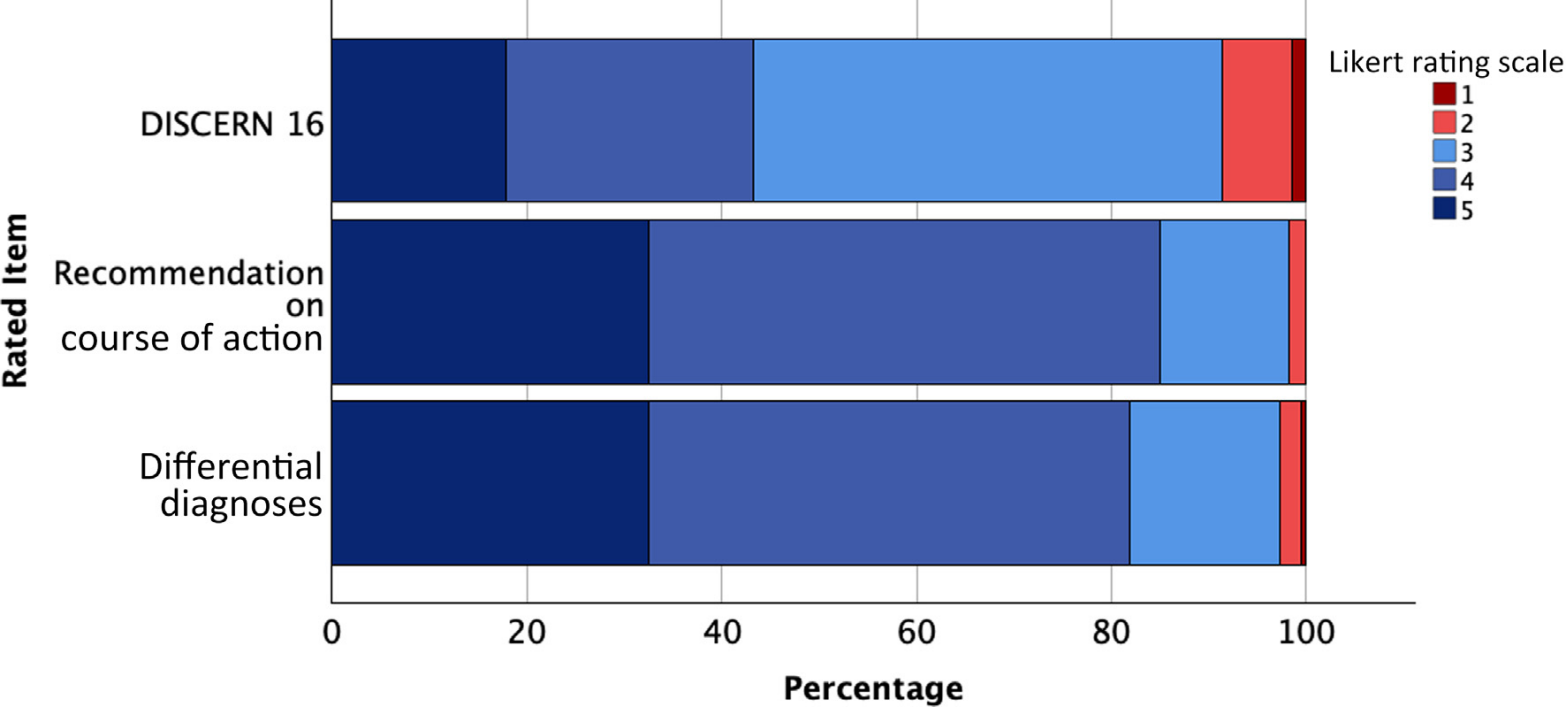
Percentage results for Likert-scale ratings for differential diagnoses, recommendations on a course of action, and DISCERN question 16 on the overall quality of the ChatGPT output. 1 = extensive shortcomings/no conformity with guidelines; 2 = important shortcomings/some conformity with guidelines; 3 = potentially important shortcomings/partial conformity with guidelines; 4 = minor shortcomings/predominant conformity with guidelines; 5 = minimal shortcomings/full conformity with guidelines.

We analyzed a total of 472 diagnostic and recommendation outputs. The GPT-3.5 outputs had median ratings of 4 (interquartile range [IQR] 4–5, range 2–5; *n* = 440) for DD, 4 (IQR 4–5, range 2–5; *n* = 440) for CoA recommendations, and 3 (IQR 3–5, range 1–5; *n* = 439) for D16 (Fig. 1).

English outputs yielded higher mean ratings for DD (4.27 vs 3.95; *p* < 0.001) and CoA (4.25 vs 4.05; *p* < 0.005) than the corresponding German outputs. There was no significant difference in mean D16 rating between English and German (3.48 vs 3.55; *p* = 0.41).

There were no significant differences between urgent (*n* = 142) and non-urgent (*n* = 298) disease patterns for DD (*p* = 0.77), CoA (*p* = 0.62), or D16 (*p* = 0.24).

**Fig. 2:**
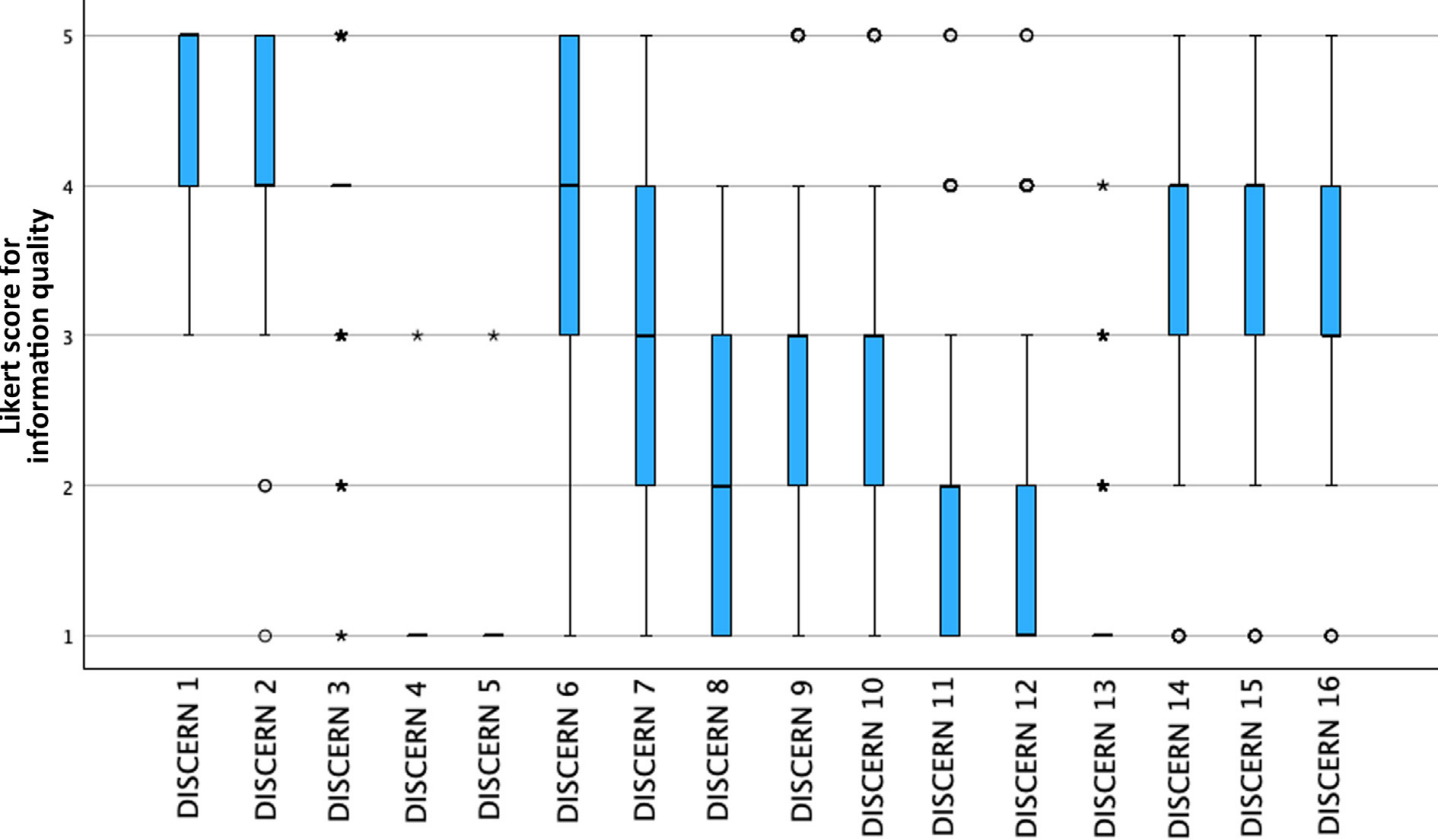
Descriptive statistics for all the DISCERN items. × = extreme outlier (interquartile range >3); ○ = mild outlier (interquartile range <3).

Analysis of information quality using DISCERN [9] revealed a wide variety among items. With a mean rating >4, item 1 (transparency of the output objectives) and item 2 (achievement of these objectives) performed best. Items 4 and 5, evaluating information sources and recency, both had a mean rating of 1 (range 1–3). Item 8 (mention of uncertainty in evidence), item 11 (description of risks associated with treatment options), item 12 (description of outcomes without treatment), and item 13 (description of the influence of the treatments on quality of life) all had a mean rating of <2 (Fig. 2).

### 3.2 IRR

The Fleiss κ values for IRR were nonsignificant for DD (κ = −0.01, *p* = 0.55), CoA (κ = −0.04, *p* = 0.12), and D16 (κ = −0.04, *p* = 0.07), corresponding to poor rater agreement [10] for all three measures.

### 3.3 Descriptive statistics

Analysis of the correlation between the rating score and the output text length in terms of the number of characters revealed no significant association for DD (*p* = 0.82) or D16 (*p* = 0.28). The correlation for CoA was also nonsignificant, but there was a weak tendency for a better rating for shorter recommendations (Spearman’s ρ = −0.07; *p* = 0.14). Analysis of the number of words instead of characters revealed a significant positive correlation for DD (Spearman’s ρ = 0.146; *p* = 0.002), with a higher word count resulting in a better DD rating.

GPT-3.5 frequently points out its own shortcomings to users with the disclaimer that it is “not a doctor”, but can offer general guidance on when to seek medical attention on the basis of the user information provided. The wording “not a doctor” was used for 228 of the 240 (95.0%) English DD outputs and 172 of the 240 (71.7%) English CoA outputs. This wording is always combined with a recommendation to “err on the side of caution and obtain proper guidance”, reminding the user that “only a healthcare professional can provide a definitive diagnosis and appropriate guidance based on […] [the] specific situation”.

The German equivalent was mentioned in 172 of the 203 (84.7%) German DD outputs and 121 of the 203 (59.6%) German CoA outputs.

Although no quantitative analysis was performed, all of the raters reported showed GPT-3.5 empathy, mainly in terms of embedding medical advice in a response targeted at the concerns and emotional stress expressed in the input. For example, GPT-3.5 picked up on financial worries mentioned in the input for one query and recommended, “Please do not delay in seeking medical assistance, even if you do not have insurance. Your health and well-being are of primary concern in this situation.”

## 4 Discussion

Our analysis shows the high potential of GPT-3.5 as an easily accessible online triage system for acute urological conditions via its better ability to react to information from individual patient in comparison to current patient information websites. English outputs were rated better than the corresponding German outputs. In addition, GPT-3.5 succeeds in applying a perceivable level of empathy when answering medical questions. Raters tended to prefer lengthy lists for DD but concise CoA recommendations.

Despite its good performance in answering patient-like questions, GPT-3.5 clearly has methodological shortcomings in terms of a lack of transparency. The occurrence of hallucinations is also an issue, whereby information is wrongly combined and extrapolated, resulting in misinformation that is made up [11]. In comparison to its performance in other specialties, GPT-3.5 still lacks detailed expert urological knowledge [12]. While this calls for broader research before implementation, the pre-triage setting appears to be the most appropriate starting point. The strain on emergency care centers in Germany caused by cases that could be managed via a lower level of care [13] could be reduced by such a pre-triage system. Current systems in use are mostly based on fixed path-finding algorithms (eg, smed pathfinder; https://smed.health/#/pathfinder/assessment).

The good technical performance of GPT-3.5 was not fully backed by good information quality. With a lack of indication of the source and the recency of the information, patients can easily be misled and are unable to verify the output. In addition, a risk assessment of therapeutic alternatives and the option of no treatment is only partly provided. However, this might be attributable to our study design, as only closed questions for a predefined CoA were posed. After describing the symptom presentation, the input did not ask for actual treatment possibilities, which were thus only roughly outlined in some outputs.

In the vast majority of cases, GPT-3.5 points out its shortcomings with a disclaimer stating that it cannot replace professional medical advice, which should be sought for reliable decision-making.

In comparison to other sources of online health information, such as videos on urolithiasis [14] or testicular cancer [15], [16], the GPT-3.5 performance for D16 was comparable or higher. Written online information on testicular cancer provided on websites with partial Health on the Internet Foundation certification [17] did not report single items, and showed only mediocre total DISCERN scores [18]. With all of these previous studies highlighting a lack of information reliability and transparency, GPT-3.5 seems to be subject to the same limitations as other online information sources, despite achieving higher absolute ratings.

High IRR has been reported for other common triage systems [19], [20], [21], in contrast to the suboptimal IRR in our study, which reduces the precision of our estimates of GPT-3.5 performance.

We measured IRR using the relatively conservative Fleiss κ metric [22]. While IRR is one measure of validity, there are several validation metrics available, and previous studies found relevant inconsistencies among the various measures [23]. A validation measure that includes assessment of the further course of events (construct validity) could be analyzed in further studies, along with a more detailed look at criterion validity to build on this the first quantitative analysis of Chat-GPT using a validated instrument (DISCERN score) and the first large scale, systematic, multirater evaluation of GPT-3.5 as a urological triage system.

Our IRR results represent an interesting finding, demonstrating not only the difficulty in measuring the quality of AI recommendations but also the existence of perceptual heterogeneity, even among professional experts with preliminary training in evaluation of AI recommendations. With a trend towards higher reliability for D16, our results emphasize agreement on the overall quality of the outputs (median D16 score of 3) but disagreement on possible diagnoses and the recommended CoA. Overall, these findings indicate that further efforts to refine and enhance the performance of GPT-3.5 are necessary.

In comparison to German outputs, English outputs received a better average rating for both DD and CoA. As the exact procedure for GPT-3.5 language processing has not been published, it is unclear whether non-English queries are directly processed or are translated to English, processed, and then re-translated back to the input language. Cross-language performance has not been systematically evaluated for GPT-3.5, but has been analyzed and reported for GPT-4 (https://openai.com/index/gpt-4/) [24]. According to this technical report, the English version of GPT-4 outperforms the German version in the accuracy of massive multitask language understanding (85.5% vs. 83.7%). Nonetheless, it is possible that raters tend to evaluate less critically in a foreign language than in their own language, resulting in bias.

The high level of empathy we observed for GPT-3.5 was previously quantified by Ayers et al [25] and mimicked a good understanding of the symptoms described by users and the associated psychological strain. Although we did not quantify this empathetic effect, it might have influenced the total rating, as no item allowed control for empathy separately. However, it has been shown that an individual and empathetic approach in patient–health care interactions increases patients’ willingness to share information pertinent for an accurate diagnosis, to follow advice, and to adhere to the prescribed treatment, and improves overall patient satisfaction [26].

While our study offers valuable insights into the capabilities of ChatGPT, it is essential to acknowledge the limitations. The method for question entry may have introduced bias. Future research should evaluate differences in ChatGPT answers at different locations, in different countries, and at different time points. Moreover, the outputs for queries entered by two of the authors were evaluated by another two authors. However, our analyses showed that IRR was limited, which probably reduces potential distortion of the results.

Regarding the languages evaluated in our study, a pertinent question is whether German is a representative language besides English. According to the GPT-4 technical report [24], English prompts provide the most accurate answers, probably because of the high volume of inputs and superior training data. Nevertheless, German ranks among the top five languages in terms of output performance. However, other common languages such as Spanish score comparably. Therefore, future evaluations should consider a wider selection of languages. In addition, new tools for assessing the competency of ChatGPT in providing accurate answers to questions are needed and should be addressed in further studies.

Finally, physicians evaluated the ChatGPT answers in our study, which may have led to some inaccuracy. Physicians’ views are subjective, and language, especially in the vague answers given by GPT-3.5, can leave room for interpretation. However, we chose to evaluate the outputs using a high number of specialist raters to mitigate this effect. Differences in interpretation between medical specialists and patients are a limitation of our study. For further assessment, a prospective study is needed. For example, the level of understanding of real patients of their diagnosis and treatment before and after receiving AI information could be evaluated using patient questionnaires.

## 5 Conclusions

In conclusion, GPT-3.5 has potential as a triage system for acute medical conditions and offers valuable insights into patient symptoms and concerns. However, information quality gaps, better transparency, and validation are needed for successful integration of the model into health care practice. Further research should explore the broader implications of ChatGPT use, considering its impact on patient outcomes, health care resource utilization, and the overall patient experience. As generative language–based AI continues to evolve, refinement and validation of applications in specific medical contexts will be crucial to ensure responsible and effective use in patient care.

  ***Author contributions***: Annemarie Uhlig had full access to all the data in the study and takes responsibility for the integrity of the data and the accuracy of the data analysis.

  *Study concept and design*: Hirtsiefer, Uhlig.

*Acquisition of data*: Hirtsiefer, Uhlig, Nestler, Eckrich, Beverungen, Siech, Aksoy, Leitsmann, Baunacke.

*Analysis and interpretation of data*: Hirtsiefer, Uhlig, Baunacke.

*Drafting of the manuscript*: Hirtsiefer, Uhlig.

*Critical revision of the manuscript for important intellectual content*: Uhlig, Baunacke, Leitsmann.

*Statistical analysis*: Hirtsiefer.

*Obtaining funding*: None.

*Administrative, technical, or material support*: Baunacke, Uhlig.

*Supervision*: Baunacke, Uhlig.

*Other*: None.

  ***Financial disclosures:*** Annemarie Uhlig certifies that all conflicts of interest, including specific financial interests and relationships and affiliations relevant to the subject matter or materials discussed in the manuscript (eg, employment/affiliation, grants or funding, consultancies, honoraria, stock ownership or options, expert testimony, royalties, or patents filed, received, or pending), are the following: None.

  ***Funding/Support and role of the sponsor*:** None.
