# Supplementary material for: Capabilities of ChatGPT-3.5 as a Urological Triage System
Source: Eur Urol Open Sci. 2024 Nov 1;70:148–53. doi: 10.1016/j.euros.2024.10.015 (PMC11567918; doi:10.1016/j.euros.2024.10.015)
Supplement: Supplementary Data 1 [file mmc1.docx]

**Supplementary material**

SupplementaryTable 1: Sources of symptom descriptions sourced from online patient fora

| **Scrotal pain** | |
| --- | --- |
| *German* | *English* |
| https://www.gutefrage.net/a/frage/hodenschmerzen-was-machen  https://www.urbia.de/forum/10-gesundheit-medizin/5592211-schmerzen-rechter-hoden  https://www.gesundheitsfrage.net/g/frage/heftige-schmerzen-an-den-hoden  https://www.gesundheitsfrage.net/g/frage/hodenschmerzen-hoden-verdrehthodentorsion | https://www.menshealthforum.org.uk/questions-pain-and-other-testicle-worries  https://www.menshealthforum.org.uk/questions-pain-and-other-testicle-worries  https://www.menshealthforum.org.uk/questions-pain-and-other-testicle-worries  https://patient.info/forums/discuss/ache-in-right-testicle-please-help-me-figure-this-out-772883  https://forum.testicularcancersociety.org/forum/before-diagnosis/do-i-have-testicular-cancer/9630-dull-ache-that-wont-go-away |
| **Testicular mass** | |
| *German* | *English* |
| https://www.gutefrage.net/a/frage/knubbel-an-hoden  https://fragen.onmeda.de/forum/männergesundheit/2029547-komische-beule-am-hoden  https://www.gesundheitsfrage.net/g/frage/habe-ich-hodenkrebs-bitte-um-schnelle-hilfe  https://www.gesundheitsfrage.net/a/frage/knoten-auf-dem-hoden  https://www.krebs-kompass.de/showthread.php?t=63480 | https://www.reddit.com/r/testicularcancer/comments/i8jiyn/lump_at_the_bottom_of_the_testicle/?rdt=60596  https://www.reddit.com/r/japanlife/comments/u96lav/so_i_just_noticed_a_lump_in_my_balls_whats_the/  https://www.reddit.com/r/AskReddit/comments/dpket/i_have_a_semihard_ball_on_my_testicle_where_do_i/  https://www.reddit.com/r/AskReddit/comments/fsc0p/felt_a_lump_on_my_testicle_anyone_have_any/  https://www.reddit.com/r/testicularcancer/comments/x3n8qw/hard_testicle/ |
| **Haematuria** | |
| *German* | *English* |
| https://www.gutefrage.net/frage/blut-im-urin-seit-tagen  https://www.gutefrage.net/frage/was-koennen-die-ursachen-fuer-blut-im-urin-sein  https://www.krebs-kompass.de/showthread.php?t=27891  https://forum.glamour.de/t/blut-im-urin-beim-mann/25441  https://fragen.lifeline.de/expertenrat/frage/Expertenrat-Urologie/Blut-im-Urin?threadId=15129769 | https://www.reddit.com/r/needadvice/comments/4frikk/serious_blood_with_my_pee_but_it_doesnt_hurt_help/  https://www.fightbladdercancer.co.uk/real-stories/craig  https://community.macmillan.org.uk/cancer_types/bladder-cancer-forum/f/general/200711/blood-in-urine-very-frightened  https://forums.runnersworld.co.uk/discussion/348405/blood-in-urine  https://www.reddit.com/r/KidneyStones/comments/6wcfum/blood_in_pee_recurring_no_pain/ |
| **Urinary Tract Infection** | |
| *German* | *English* |
| https://www.gesundheitsfrage.net/g/frage/komische-schmerzen-beim-wasserlassen-urinieren  https://fragen.onmeda.de/forum/gynäkologie/3209141-seit-3-wochen-brennen  https://www.gesundheitsfrage.net/g/frage/unterleibschmerzen-und-farbloser-urin  https://www.gesundheitsfrage.net/g/frage/nierenschmerzen-und-staendiger-harndrang  https://www.gesundheitsfrage.net/frage/staendiger-harndrang-2 | https://www.reddit.com/r/NoStupidQuestions/comments/3kkl2b/guys_does_anyone_else_get_that_mild_un/  https://www.reddit.com/r/AskReddit/comments/wvzuw/hey_reddit_it_really_hurts_to_pee_and_i_cant_see/  https://www.reddit.com/r/endometriosis/comments/dqifqh/pain_while_peeing_could_it_be_endometriosis/  https://www.reddit.com/r/TwoXChromosomes/comments/ozmhp0/kidney_infection/  https://www.reddit.com/r/TwoXChromosomes/comments/14ks3p6/dealing_with_my_first_uti_im_getting_really/ |
| **Urolithiasis** | |
| *German* | *English* |
| https://www.gutefrage.net/frage/nierenschmerzen-ohne-andere-symptome  https://www.gesundheitsfrage.net/frage/schmerzen-linke-flanke-muskulatur-oder-nieren  https://www.gesundheitsfrage.net/frage/blasenentzuendung-negativ-nieren-schmerzen-wie-die-hoelle  https://www.gesundheitsfrage.net/frage/unerklaerliche-schmerzen-im-bereich-der-rechten-rippen-kann-jemand-helfen  https://blog.patrickkempf.de/2006/ciao-nierenstein/ | https://patient.info/forums/discuss/stones-inside-the-kidney--529168  https://www.reddit.com/r/KidneyStones/comments/u36h2p/severe_pain_in_right_flank_when_moving_feels_like/  https://www.reddit.com/r/KidneyStones/comments/fydidl/intermittent_flank_pain_kidney_stone_or_something/  https://www.reddit.com/r/KidneyStones/comments/11hwege/flank_pain_for_months/  https://www.reddit.com/r/KidneyStones/comments/e4if22/stabbing_but_intermittent_pain_beginning_of/ |
| **Priapism** | |
| *German* | *English* |
| https://www.gutefrage.net/frage/staender-geht-nicht-weg-w  https://www.extrem-bodybuilding.de/forum/thread/42671-viagra-wirkung-will-nicht-weg/  https://www.joyclub.de/groups/keuschhaltung/forum/t1518386.dauerstaender_priapismus.html  https://www.erektion.de/t/schmerzhafte-naechtliche-dauererektion.1080/  https://www.erektion.de/t/schmerzhafte-naechtliche-dauererektion.1080/ | https://www.reddit.com/r/TooAfraidToAsk/comments/au2ek3/men_who_have_had_a_priapism_how_are_you_and_how/  https://www.reddit.com/r/Advice/comments/o8hn79/my_dick_wont_go_soft/  https://www.reddit.com/r/NoStupidQuestions/comments/nxijyb/can_having_a_erection_for_long_periods_of_time_be/  https://patient.info/forums/discuss/painful-erection-284393  https://www.reddit.com/r/LSD/comments/t7126l/i_had_lsdinduced_priapism_an_erection_lasting_4/ |

Table 2: Exemplary German inputs and corresponding outputs generated by GPT-3.5

| **Symptom** | **Input** | **Output 1** | **Follow-Up Input** | **Output 2** |
| --- | --- | --- | --- | --- |
| Testicular pain (German) | Also meine Schmerzen kann ich in etwa so beschreiben, wie wenn ich einen Tritt in die Eier kassiert hätte. Das zieht auch so in den Bauch rein! Genau so ein Ziehen empfinde ich und dazu kommt, dass meine Eier sehr berührungsempfindlich. Anfassen = Schmerzen. Ich habe mir schon überlegt, ob ich mir die Hoden verdreht habe, aber ich will das nicht meinen Eltern sagen. Schmerzen habe ich seit heute sehr sehr stark, vor zwei Tagen ist es das erste Mal leicht aufgetreten  Was könnte das sein? Bitte zähle die möglichen Krankheitsbilder auf! | Ich bin kein Arzt, aber ich kann versuchen, Ihnen einige mögliche Ursachen für Ihre Beschwerden zu nennen. Es ist wichtig zu beachten, dass eine genaue Diagnose nur von einem medizinischen Fachmann gestellt werden kann. Wenn Sie starke Schmerzen haben, sollten Sie unbedingt einen Arzt aufsuchen, um die Ursache Ihrer Symptome abzuklären. Hier sind einige mögliche Krankheitsbilder, die ähnliche Symptome verursachen könnten:  1. Hodentorsion: Eine Hodentorsion tritt auf, wenn sich der Hoden verdreht und dadurch die Blutversorgung abgeschnitten wird. Dies kann starke Schmerzen im Hoden verursachen und erfordert sofortige medizinische Hilfe.  2. Hodenentzündung (Orchitis): Orchitis ist eine Entzündung des Hodens, die Schmerzen und Schwellungen verursachen kann. Es tritt häufig als Komplikation einer Infektion wie Mumps oder einer Harnwegsinfektion auf.  3. Nebenhodenentzündung (Epididymitis): Die Epididymitis ist eine Entzündung des Nebenhodens, die meist durch eine bakterielle Infektion verursacht wird. Sie kann zu Schmerzen im Hoden führen und tritt häufig in Verbindung mit einer Harnwegsinfektion auf.  4. Inguinalhernie: Eine Inguinalhernie tritt auf, wenn Gewebe, zum Beispiel Teile des Darms, durch eine Schwachstelle in der Bauchwand in den Leistenkanal rutscht. Dies kann zu Schmerzen in den Hoden führen.  5. Hodenverletzung: Eine Verletzung des Hodens, wie zum Beispiel ein Schlag oder Stoß, kann starke Schmerzen verursachen. Eine Hodenverletzung erfordert ebenfalls medizinische Untersuchung und Behandlung.  Diese Liste ist nicht abschließend, und es gibt noch weitere mögliche Ursachen für Ihre Beschwerden. Wenden Sie sich bitte an einen Arzt, um eine genaue Diagnose und angemessene Behandlung zu erhalten. Es ist wichtig, dass Sie Ihren Eltern von Ihren Schmerzen erzählen, damit sie Ihnen bei einem Arztbesuch helfen können. | Würdest Du mir anhand dessen jetzt empfehlen mich sofort in der Notaufnahme vorzustellen, morgen beim Hausarzt oder im Laufe der Woche beim Urologen? | Als KI-Modell bin ich nicht in der Lage, eine persönliche medizinische Beratung durchzuführen, da ich keine Informationen über Ihre individuelle Situation habe. Ich kann Ihnen jedoch allgemeine Ratschläge geben. Da Sie Schmerzen und Empfindlichkeit im Hoden haben, ist es ratsam, die Symptome ernst zu nehmen und einen medizinischen Fachmann aufzusuchen. Wenn der Schmerz stark ist und sich verschlimmert, könnte dies auf eine akute Erkrankung wie eine Hodentorsion hinweisen, die einen medizinischen Notfall darstellt. In einem solchen Fall sollten Sie sich sofort in der Notaufnahme vorstellen. Wenn der Schmerz erträglich ist und sich nicht verschlimmert, können Sie erwägen, morgen einen Termin bei Ihrem Hausarzt zu vereinbaren. Der Hausarzt kann Ihre Symptome bewerten und Sie bei Bedarf an einen Urologen überweisen. Es ist wichtig, dass Sie die Meinung eines medizinischen Fachmanns einholen, um eine genaue Diagnose zu erhalten und eine angemessene Behandlung zu erhalten. Sie können auch den ärztlichen Bereitschaftsdienst kontaktieren, um eine Einschätzung der Dringlichkeit Ihrer Symptome zu erhalten. Bitte beachten Sie, dass dies allgemeine Ratschläge sind und keine persönliche medizinische Beratung ersetzen können. Konsultieren Sie immer einen Arzt, um Ihre spezifische Situation zu bewerten. |
|  |  |  |  |  |
| Testicular mass (German) | Es ist mir etwas unangenehm darüber zu sprechen aber es muss sein.. und zwar bin ich 17 Jahre alt und seit 1 oder 2 Jahren maximal hab ich gemerkt, dass mein rechter Hoden anders ist, als der andere. Beschreibung: Zuerst mal fühlt es sich an als hätte ich einen dritten Hoden. Und zwar beim rechten Hoden ist noch irgendwas Grösseres dran, was fast genauso groß ist wie der Hoden selbst. (Schwer zu erklären). Das ist nicht getrennt vom Hoden sondern aneinander.  Was könnte das sein? Bitte zähle die möglichen Krankheitsbilder auf!° | Ich verstehe, dass es unangenehm sein kann, darüber zu sprechen, aber es ist wichtig, deine Gesundheit ernst zu nehmen. Es gibt einige mögliche medizinische Zustände, die die Veränderung in deinem Hoden verursachen könnten. Bitte beachte jedoch, dass ich kein Arzt bin und keine genaue Diagnose stellen kann. Es ist wichtig, einen Arzt aufzusuchen, um eine professionelle Untersuchung und Diagnose zu erhalten. Einige mögliche Ursachen könnten sein:  1. **Varikozele**: Dies ist eine Erweiterung der Venen im Hodensack. Es kann zu einem Gefühl von "drittem Hoden" führen, da die Venen manchmal geschwollen wirken können.  2. **Hernie**: Eine Hernie tritt auf, wenn ein Teil des Darms oder des Bauchfells durch eine Schwachstelle in der Bauchwand rutscht. Dies könnte zu einer Veränderung in der Größe oder Form des Hodensacks führen.  3. **Hydrozele**: Eine Ansammlung von Flüssigkeit um den Hoden kann zu einer Vergrößerung führen und das Gefühl eines zusätzlichen Teils im Hodensack vermitteln.  4. **Epididymitis oder Orchitis**: Entzündungen des Nebenhodens (Epididymitis) oder des Hodens selbst (Orchitis) können Schwellungen und Veränderungen in der Form verursachen.  5. **Tumor oder Zyste**: Tumore oder Zysten im Hoden könnten zu einer unregelmäßigen Größenzunahme oder Veränderung führen.  6. **Andere Erkrankungen**: Es gibt auch andere seltene Erkrankungen oder Anomalien, die zu Veränderungen im Hodenbereich führen könnten. Es ist wirklich wichtig, dass du dich von einem Arzt untersuchen lässt, um eine genaue Diagnose zu erhalten. Du könntest zunächst deinen Hausarzt oder einen Urologen aufsuchen. Je früher du medizinischen Rat einholst, desto besser können mögliche Probleme behandelt werden. | Würdest Du mir anhand dessen jetzt empfehlen mich sofort in der Notaufnahme vorzustellen, morgen beim Hausarzt oder im Laufe der Woche beim Urologen? | Ich bin kein Arzt, aber ich kann dir einige allgemeine Ratschläge geben. In Situationen, in denen du Veränderungen an deinem Körper bemerkst, insbesondere im Bereich der Genitalien, ist es wichtig, deine Gesundheit ernst zu nehmen und medizinische Hilfe in Anspruch zu nehmen. Da du bereits seit 1 oder 2 Jahren Veränderungen an deinem Hoden bemerkst und du besorgt bist, wäre es wahrscheinlich ratsam, so bald wie möglich ärztliche Hilfe in Anspruch zu nehmen. Ob du dich sofort in der Notaufnahme vorstellst oder einen Termin beim Hausarzt oder Urologen vereinbarst, hängt von verschiedenen Faktoren ab:  1. **Schmerzen oder akute Beschwerden**: Wenn du Schmerzen oder akute Beschwerden im Zusammenhang mit den Veränderungen hast, könntest du in die Notaufnahme gehen, um sicherzustellen, dass es sich nicht um eine ernsthafte sofortige medizinische Angelegenheit handelt.  2. **Keine akuten Beschwerden**: Wenn du keine starken Schmerzen oder dringenden Beschwerden hast, könntest du in Erwägung ziehen, einen Termin beim Hausarzt oder Urologen zu vereinbaren. Dies könnte dir die Gelegenheit geben, deine Situation in einem weniger eiligen Umfeld zu besprechen.  3. **Verfügbarkeit von Terminen**: Je nachdem, wie schnell du einen Termin beim Hausarzt oder Urologen bekommen kannst, könnte dies auch eine Rolle bei deiner Entscheidung spielen.  Es ist jedoch wichtig zu betonen, dass dies allgemeine Ratschläge sind und keine medizinische Beratung ersetzen. Wenn du besorgt bist oder Unsicherheiten bestehen, solltest du auf jeden Fall professionellen medizinischen Rat einholen. Es könnte hilfreich sein, deinen Eltern oder einem vertrauten Erwachsenen von deinen Bedenken zu erzählen, damit sie dich unterstützen können, die beste Entscheidung für deine Gesundheit zu treffen. |
|  |  |  |  |  |
| Haematuria (German) | Hallo ich habe seit 5-6 Tagen Blut im Urin. Das macht mir schreckliche sorgen.Ich war beim Arzt und die haben mir gesagt dass sie es Untersuchen.  Was könnte das sein? Bitte zähle die möglichen Krankheitsbilder auf! | Es ist wichtig zu betonen, dass ich kein Arzt bin, aber ich kann Ihnen einige mögliche Ursachen für Blut im Urin nennen. Blut im Urin, medizinisch als "Hämaturie" bezeichnet, kann auf verschiedene gesundheitliche Probleme hinweisen. Die genaue Ursache kann nur von einem qualifizierten Arzt nach gründlicher Untersuchung und Diagnose festgestellt werden.  Hier sind einige mögliche Krankheitsbilder, die Blut im Urin verursachen könnten:  Harnwegsinfektion (HWI): Eine bakterielle Infektion der Harnwege kann Entzündungen verursachen, die zu Blut im Urin führen können.  Nierensteine: Kleine Kristalle können sich in den Nieren bilden und beim Ausscheiden durch die Harnwege Blutungen verursachen.  Blasenentzündung: Eine Entzündung der Blasenwand kann zu Blutungen führen.  Verletzungen oder Trauma: Ein Schlag oder eine Verletzung in der Nähe der Harnwege kann Blut im Urin verursachen.  Nierenkrankheiten: Verschiedene Nierenerkrankungen wie Glomerulonephritis können Blut im Urin verursachen.  Prostataprobleme: Bei Männern können Erkrankungen der Prostata, wie eine Prostatitis oder ein Prostataadenom, Blut im Urin verursachen.  Blutgerinnungsstörungen: Einige Erkrankungen, die zu einer gestörten Blutgerinnung führen, können Blutungen in den Harnwegen verursachen.  Harnwegsverengungen: Engstellen oder Verengungen in den Harnwegen können die Durchblutung beeinträchtigen und Blutungen verursachen.  Krebserkrankungen: Bösartige Tumoren in den Harnwegen, den Nieren, der Blase oder der Prostata können Blut im Urin verursachen.  Medikamente: Bestimmte Medikamente können als Nebenwirkung Blutungen verursachen.  Schwere körperliche Anstrengung: Extrem intensive körperliche Aktivität könnte zu vorübergehenden Blutungen führen.  Es ist wichtig, dass Sie die Untersuchungsergebnisse und die Diagnose Ihres Arztes abwarten. Falls das Problem länger anhält oder sich verschlimmert, sollten Sie auf jeden Fall sofort ärztliche Hilfe in Anspruch nehmen. Nur ein Facharzt kann eine genaue Diagnose stellen und die entsprechende Behandlung empfehlen. | Würdest Du mir anhand dessen jetzt empfehlen mich sofort in der Notaufnahme vorzustellen, morgen beim Hausarzt oder im Laufe der Woche beim Urologen? | Ich kann keine direkte medizinische Beratung oder Empfehlungen geben, da ich keine persönlichen medizinischen Informationen habe und kein Arzt bin. Allerdings kann ich Ihnen einige allgemeine Hinweise geben:  Blut im Urin kann auf verschiedene ernsthafte medizinische Probleme hinweisen, einschließlich solcher, die sofortige Aufmerksamkeit erfordern. Wenn Sie starke Schmerzen, Fieber, Schüttelfrost oder Schwierigkeiten beim Wasserlassen haben, sollten Sie umgehend ärztliche Hilfe in Anspruch nehmen. In solchen Fällen wäre der Gang in die Notaufnahme gerechtfertigt.  Wenn die Symptome milder sind und keine akute Gefahr für Ihre Gesundheit besteht, könnte es angemessen sein, zunächst einen Termin beim Hausarzt zu vereinbaren. Der Hausarzt kann eine erste Beurteilung durchführen und Sie gegebenenfalls an einen Facharzt, wie einen Urologen, überweisen.  Die genaue Empfehlung hängt von der Schwere Ihrer Symptome, Ihrem allgemeinen Gesundheitszustand und anderen individuellen Faktoren ab.  Wenn Sie sich unsicher sind, ist es ratsam, medizinischen Rat von einem Fachmann einzuholen, entweder telefonisch oder persönlich. In jedem Fall ist es wichtig, Ihre Symptome ernst zu nehmen und ärztlichen Rat einzuholen, um die Ursache für das Blut im Urin festzustellen und angemessen behandeln zu lassen. |
|  |  |  |  |  |
| Urinary tract infection (German) | Hallo. Ich war vorhin auf der Toilette und musste Komische schmerzen beim Harnlassen feststellen. Immer wenn ich das gefühl habe ich bin fertig sticht es so leicht bei der eichel und das ist sehr unangenehm.  Was könnte das sein? Bitte zähle die möglichen Krankheitsbilder auf! | Ich bin kein Arzt, aber ich kann versuchen, einige mögliche Ursachen für die von Ihnen beschriebenen Symptome zu nennen. Es ist wichtig zu beachten, dass nur ein qualifizierter medizinischer Fachmann nach einer Untersuchung eine genaue Diagnose stellen kann. Hier sind einige mögliche Krankheitsbilder, die Schmerzen oder Beschwerden beim Harnlassen verursachen könnten:  1. **Harnwegsinfektion (HWI):** Eine Infektion der Harnwege, einschließlich der Blase, kann zu Schmerzen und Brennen beim Harnlassen führen.  2. **Harnröhrenentzündung (Urethritis):** Eine Entzündung der Harnröhre kann ähnliche Symptome wie eine Harnwegsinfektion verursachen.  3. **Sexuell übertragbare Infektionen (STIs):** STIs wie Gonorrhoe oder Chlamydien können zu Harnröhrenentzündungen führen und Schmerzen beim Wasserlassen verursachen.  4. **Prostatitis:** Eine Entzündung der Prostata kann zu Schmerzen im Genitalbereich, einschließlich der Eichel, führen.  5. **Harnstein:** Ein Harnstein, der sich in der Harnröhre oder in der Blase befindet, kann Schmerzen oder Irritationen beim Harnlassen verursachen.  6. **Phimose:** Eine Verengung der Vorhaut kann zu Schmerzen führen, insbesondere beim Zurückschieben der Vorhaut.  7. **Allergische Reaktion:** Eine allergische Reaktion auf Seife, Waschmittel oder andere Substanzen im Genitalbereich kann zu Irritationen und Schmerzen führen.  8. **Verletzungen:** Verletzungen im Genitalbereich können Schmerzen und Unbehagen beim Wasserlassen verursachen.  9. **Reizung:** Reizungen durch enge Kleidung, aggressive Hygienepraktiken oder andere Faktoren können zu Beschwerden führen.  Da die Ursachen von Schmerzen beim Harnlassen vielfältig sein können, ist es wichtig, einen Arzt aufzusuchen, um eine genaue Diagnose zu erhalten. Ein Arzt kann Ihre Symptome beurteilen, Sie untersuchen und gegebenenfalls Tests durchführen, um die genaue Ursache festzustellen und die angemessene Behandlung zu empfehlen. | Würdest Du mir anhand dessen jetzt empfehlen mich sofort in der Notaufnahme vorzustellen, morgen beim Hausarzt oder im Laufe der Woche beim Urologen? | Ich bin kein Arzt, kann also keine individuellen medizinischen Ratschläge geben. Allerdings basierend auf den Informationen, die Sie mir gegeben haben, gibt es einige Faktoren zu berücksichtigen:  1. **Schwere der Symptome:** Wenn die Schmerzen beim Harnlassen sehr intensiv sind, begleitet von Fieber, Blut im Urin oder anderen schweren Symptomen, könnte es sinnvoll sein, sofort medizinische Hilfe in der Notaufnahme zu suchen.  2. **Dringlichkeit:** Wenn die Schmerzen leicht bis moderat sind und keine schweren Begleitsymptome auftreten, könnten Sie eventuell bis morgen warten, um Ihren Hausarzt aufzusuchen.  3. **Falls bekannt, Vorgeschichte:** Wenn Sie bereits in der Vergangenheit Probleme mit dem Harnsystem hatten oder eine bekannte Krankheitsgeschichte haben, könnte eine Untersuchung beim Urologen in Erwägung gezogen werden. In jedem Fall ist es ratsam, bei anhaltenden oder sich verschlechternden Symptomen einen medizinischen Fachmann aufzusuchen.  Wenn Sie sich unsicher sind, können Sie auch versuchen, den ärztlichen Bereitschaftsdienst oder eine medizinische Hotline anzurufen, um Rat einzuholen. Ihr Wohlbefinden hat oberste Priorität, daher sollten Sie die Option wählen, die Ihnen am sinnvollsten erscheint, um eine angemessene medizinische Versorgung zu erhalten. |
|  |  |  |  |  |
| Urolithiasis (German) | Moin, ich hab seit 2 Tagen wieder starke Nierenschmerzen aber ohne andere Symptome wie Fieber etc. also es geht mir abgesehen von den Schmerzen körperlich super. Ich hatte das vor ca 3 Wochen schon mal mit einer Blasenentzündung zusammen, war auch beim Arzt. Die hat mir nur antibiotika gegen die Blasenentzündung gegeben und die war am nächsten tag auch weg, aber meine Nierenschmerzen immer noch stark da.  Was könnte das sein? Bitte zähle die möglichen Krankheitsbilder auf! | Ich bin kein Arzt, aber ich kann dir einige mögliche Ursachen für starke Nierenschmerzen nennen. Es ist jedoch wichtig, dass du ärztlichen Rat einholst, um eine genaue Diagnose und angemessene Behandlung zu erhalten. Hier sind einige mögliche Krankheitsbilder, die mit Nierenschmerzen in Verbindung stehen könnten:  Nierensteine: Kleine, harte Ablagerungen in den Nieren können starke Schmerzen verursachen, die oft in den Rücken oder die Flanken ausstrahlen. Sie können auch Übelkeit und Erbrechen verursachen.  Niereninfektion (Pyelonephritis): Dies ist eine ernsthafte Infektion der Nieren, die neben Schmerzen auch Fieber, Schüttelfrost und allgemeines Unwohlsein verursachen kann.  Nierenentzündung (Glomerulonephritis): Eine Entzündung der Nierenfilter kann zu Schmerzen, Blut im Urin, Schwellungen und Bluthochdruck führen.  Nierenabszess: Eine Ansammlung von Eiter in der Niere kann Schmerzen und Fieber verursachen.  Nierenzysten: Flüssigkeitsgefüllte Säcke in den Nieren können in einigen Fällen Schmerzen verursachen, insbesondere wenn sie groß oder symptomatisch sind.  Muskelverspannungen oder Verletzungen: Manchmal können Rückenschmerzen auf Muskelverspannungen oder Verletzungen zurückzuführen sein, die in den Nierenbereich ausstrahlen.  Lumbaler Bandscheibenvorfall: Ein Bandscheibenvorfall in der Lendenwirbelsäule kann Nerven im Bereich der Nieren beeinträchtigen und Schmerzen verursachen.  Interstitielle Nephritis: Eine Entzündung des Nierengewebes kann zu Schmerzen und verminderter Nierenfunktion führen.  Reizung der Harnwege: Eine anhaltende Reizung der Harnwege, wie eine nicht behandelte Blasenentzündung, kann zu Nierenschmerzen führen.  Andere Erkrankungen: Es gibt auch andere weniger häufige Erkrankungen, die zu Nierenschmerzen führen können, wie z.B. Tumoren oder angeborene Anomalien.  Da du bereits Nierenschmerzen hattest und diese sich nicht verbessert haben, wäre es ratsam, einen Arzt aufzusuchen, um eine genaue Diagnose und geeignete Behandlung zu erhalten. Nierenschmerzen sollten nicht leicht genommen werden, da sie auf ernsthafte Gesundheitsprobleme hinweisen können. | Würdest Du mir anhand dessen jetzt empfehlen mich sofort in der Notaufnahme vorzustellen, morgen beim Hausarzt oder im Laufe der Woche beim Urologen? | Da ich kein Arzt bin, kann ich keine medizinischen Ratschläge geben. Allerdings basierend auf den Informationen, die du mir gegeben hast, ist es ratsam, in einer Situation wie dieser, ärztlichen Rat einzuholen.  Wenn du starke Nierenschmerzen hast, die seit mehreren Tagen anhalten, könnte dies auf ein ernstes Problem hinweisen.  Wenn du unsicher bist, wäre es möglicherweise am besten, dich so bald wie möglich ärztlich untersuchen zu lassen.  Die Entscheidung, ob du sofort in die Notaufnahme gehst, dich morgen beim Hausarzt vorstellst oder einen Termin beim Urologen vereinbarst, hängt von verschiedenen Faktoren ab, wie der Schwere deiner Schmerzen, deiner persönlichen Gesundheitsgeschichte und den verfügbaren Ressourcen in deinem Gesundheitssystem. Wenn die Schmerzen sehr intensiv sind oder du zusätzliche Symptome wie Fieber oder Übelkeit hast, könnte ein Besuch in der Notaufnahme angebracht sein. Ansonsten könntest du morgen deinen Hausarzt kontaktieren und nach seinem Rat fragen. Er kann dir basierend auf deiner Krankengeschichte und den aktuellen Symptomen Empfehlungen geben.  In jedem Fall ist es wichtig, ärztlichen Rat einzuholen, um die genaue Ursache deiner Nierenschmerzen festzustellen und die entsprechende Behandlung zu erhalten. |
|  |  |  |  |  |
| Priapism (German) | Hab mal das viagra von Gencimed ausprobiert. Echt super und hatte die nacht viel spass aber mein freund da unten will nicht abschwellen. Ich will jetzt trainieren gehen aber kann doch nicht mit nem Dauerstaender rumrennen. Was kann ich machen ausser ins kalte wasser halten? Also ich hab nur eine Kapsel eingenommen (100mg sildenafil citrate laut der Packung) und das war aber schon um 00.00 also sprich 11 Stunden her.  Was könnte das sein? Bitte zähle die möglichen Krankheitsbilder auf! | Ich bin kein Arzt, aber ich kann versuchen, dir einige Informationen zu geben, die dir helfen könnten. Wenn du nach der Einnahme von Viagra (Sildenafil) immer noch eine anhaltende Erektion hast, die länger als vier Stunden dauert (auch als Priapismus bekannt), solltest du sofort ärztliche Hilfe in Anspruch nehmen.  Ein Priapismus ist ein medizinischer Notfall, da er zu schweren gesundheitlichen Komplikationen führen kann, wenn er nicht behandelt wird. Mögliche Ursachen für einen Priapismus können sein:  1. **Nebenwirkungen von Medikamenten:** Viagra (Sildenafil) ist bekannt für seine Fähigkeit, die Durchblutung zu erhöhen. In seltenen Fällen kann dies zu einer anhaltenden Erektion führen.  2. **Überdosierung:** Es ist möglich, dass eine höhere als empfohlene Dosis zu einer anhaltenden Erektion führt.  3. **Unterliegende Gesundheitszustände:** Priapismus kann auch mit bestimmten medizinischen Zuständen wie Sichelzellanämie, Leukämie oder neurologischen Erkrankungen in Verbindung gebracht werden.  4. **Wechselwirkungen mit anderen Medikamenten:** Wenn du andere Medikamente einnimmst, insbesondere solche, die mit Sildenafil interagieren könnten, kann dies zu unerwünschten Nebenwirkungen führen.  Da du bereits 11 Stunden nach der Einnahme von Viagra immer noch eine Erektion hast, solltest du sofort ärztliche Hilfe suchen. Gehe zu einem Arzt oder in ein Krankenhaus, um deine Situation zu besprechen und die notwendige Behandlung zu erhalten. Ein Fachmann wird in der Lage sein, die genaue Ursache deiner anhaltenden Erektion zu bestimmen und angemessene Maßnahmen zu ergreifen, um sie zu behandeln.  Es ist wichtig zu betonen, dass medizinische Ratschläge von einem Facharzt eingeholt werden sollten. Wenn du Fragen zu Medikamenten oder Gesundheitszuständen hast, ist es immer am besten, professionelle medizinische Hilfe in Anspruch zu nehmen. | Würdest Du mir anhand dessen jetzt empfehlen mich sofort in der Notaufnahme vorzustellen, morgen beim Hausarzt oder im Laufe der Woche beim Urologen? | Ich bin kein Arzt, aber wenn du seit 11 Stunden eine anhaltende Erektion hast, könnte dies auf einen Priapismus hinweisen, der eine medizinische Notfallsituation sein kann.  Es wird empfohlen, dass du so schnell wie möglich ärztliche Hilfe suchst. Priapismus kann zu ernsthaften Komplikationen führen, wenn er nicht rechtzeitig behandelt wird. Da es sich um eine möglicherweise dringende Situation handelt, könnte ein Besuch in der Notaufnahme gerechtfertigt sein.  Die Ärzte dort sind in der Regel besser in der Lage, akute medizinische Probleme zu bewerten und zu behandeln. Wenn du unsicher bist, ist es immer besser, auf Nummer sicher zu gehen und medizinische Fachkräfte um Rat zu bitten.  Bitte beachte, dass ich keine persönlichen medizinischen Ratschläge geben kann. Wenn du Bedenken hinsichtlich deiner Gesundheit hast, ist es am besten, professionelle medizinische Hilfe in Anspruch zu nehmen. |

Appendix Table 2: Showing one exemplary input for each of the six symptomologies into GPT-3.5. Inputs were derived from German online fora combined with the last paragraph asking for differential diagnoses. See Appendix Table 1 for sources.

Table 3: Exemplary English inputs and corresponding outputs generated by GPT-3.5

| **Symptom** | **Input** | **Output 1** | **Follow-Up Input** | **Output 2** |
| --- | --- | --- | --- | --- |
| Testicular pain (English) | My partner woke up on Wednesday night with severe scrotal/testicular pain and pain in the lower abdomen and groin. He was also being violently sick.  What could this be? Please name all the possible diagnoses! | I'm not a doctor, but I can provide some general information on possible causes of severe scrotal/testicular pain, lower abdomen pain, and groin pain, along with vomiting. These symptoms could be indicative of several different conditions, and a medical evaluation by a healthcare professional is essential for an accurate diagnosis. Here are some potential causes:  1. Testicular Torsion: This is a medical emergency in which the spermatic cord twists, cutting off blood flow to the testicle. It leads to severe testicular pain and requires immediate surgical intervention.  2. Epididymitis: Inflammation of the epididymis, a coiled tube located near the testicle, can cause testicular pain and is often accompanied by lower abdominal and groin discomfort.  3. Kidney Stones: Sometimes, kidney stones can cause referred pain that is felt in the lower abdomen and groin area. Nausea and vomiting can also occur due to the intense pain.  4. Hernia: An inguinal hernia, which occurs when a portion of the intestine pushes through the abdominal wall or into the inguinal canal, can cause lower abdominal and groin pain.  5. Orchitis: Inflammation of the testicle, often due to infection, can lead to testicular pain and discomfort.  6. Appendicitis: While the appendix is located in the lower right abdomen, the pain can sometimes radiate to the groin area.  7. Gastrointestinal Issues: Some gastrointestinal problems, such as gastritis, gastroenteritis, or bowel obstructions, can cause abdominal pain and vomiting.  8. Pelvic Inflammatory Disease (PID): In women, PID can lead to lower abdominal pain, and the pain may be felt in the groin area.  9. Other Infections: Various infections, such as urinary tract infections (UTIs) or sexually transmitted infections (STIs), may cause lower abdominal and groin pain.  10. Abdominal or Testicular Trauma: Injury to the abdomen or testicles can result in pain and discomfort.  11. Other Medical Conditions: Other conditions, such as appendicitis, diverticulitis, or even a heart attack, can cause referred pain that may be felt in the lower abdomen and groin area.  Given the severity of the symptoms, it's crucial for your partner to seek immediate medical attention. A healthcare professional will perform a physical examination and order any necessary tests to determine the cause of the symptoms and provide appropriate treatment. If there is any doubt or if the pain is severe and sudden, consider going to the nearest emergency room or calling 911 for assistance. | Given the previous information, would you suggest presenting in the Emergency Department immediately, at the GP office tomorrow or at the office of a urology consultant within the next week? | I'm not a doctor, but I can offer some general guidance based on the symptoms you've described.  If your partner is experiencing severe scrotal/testicular pain, lower abdominal pain, groin pain, and vomiting, it is essential to take these symptoms seriously. Testicular pain can sometimes be a sign of a medical emergency, especially if it's related to conditions like testicular torsion or acute epididymitis.  Given the severity of the symptoms, it's advisable to seek immediate medical attention. The Emergency Department (ED) is typically the most appropriate place to go in situations where there is severe pain and vomiting, as they can provide rapid evaluation and any necessary diagnostic tests. In cases of testicular torsion or other serious conditions, timely treatment is critical to prevent complications. If your partner's symptoms are less severe or seem to improve, it may be reasonable to schedule an appointment with a GP for further evaluation.  However, if the symptoms persist or worsen, or if there is any doubt about the seriousness of the condition, it is still recommended to visit the ED or consult with a healthcare professional as soon as possible.  Ultimately, it's best to err on the side of caution when dealing with severe pain and abdominal discomfort, as some conditions can be time-sensitive and require prompt medical attention. A healthcare professional can make the most appropriate assessment based on the specific details of your partner's condition. |
|  |  |  |  |  |
| Testicular mass (English) | I was taking a shower, and went to clean my balls. I usually just do a quick couple swipes and that's it. This time I felt a lump during one of my swipes. I went back to reexamine it and it's a hard lump about half the size of a skittle directly on my right testicle. I'm 20 years old if it matters.  What could this be? Please name all the possible diagnoses! | I'm not a doctor, but I can offer some general information that might help you. If you've discovered a lump on your testicle, it's important to take it seriously and consult with a healthcare professional as soon as possible. There are several possible diagnoses for a testicular lump, some of which include:  1. Epididymal cyst: These are benign fluid-filled sacs that can develop in the epididymis, a small organ located near the testicle. They are generally harmless.  2. Spermatocele: Similar to an epididymal cyst, a spermatocele is a cyst filled with a milky fluid that often forms on the epididymis. They are usually non-cancerous.  3. Hydrocele: A hydrocele is a buildup of fluid around the testicle, causing swelling. It is typically benign and can be caused by various factors.  4. Varicocele: This is an enlargement of the veins within the scrotum, similar to varicose veins. Varicoceles can lead to discomfort but are not cancerous.  5. Testicular torsion: This is a medical emergency in which the spermatic cord becomes twisted, cutting off blood flow to the testicle. It requires immediate medical attention.  6. Testicular cancer: Although less likely, a lump on the testicle can be a sign of testicular cancer. Testicular cancer typically affects young to middle-aged men and is highly treatable, especially when caught early.  It's crucial to see a healthcare professional, preferably a urologist, for a proper evaluation. They will likely perform a physical examination and may order additional tests, such as an ultrasound, to determine the nature of the lump. Early detection and diagnosis are essential, especially if the lump turns out to be testicular cancer. Don't hesitate to seek medical attention to address your concerns and ensure your well-being. | Given the previous information, would you suggest presenting in the Emergency Department immediately, at the GP office tomorrow or at the office of a urology consultant within the next week? | I'm not a doctor, but I can offer some general guidance. The urgency of your medical evaluation depends on several factors, including the severity of your symptoms and the level of concern you have. Here's a general guideline:  1. Emergency Department : If you experience sudden and severe pain, testicular swelling, or any other alarming symptoms, or if you suspect testicular torsion, it's essential to seek immediate medical attention at the emergency department.  2. GP Office : If you don't have severe symptoms or are not in significant pain, you can schedule an appointment with your general practitioner (GP) for the next available time. Your GP can provide an initial evaluation and then refer you to a urologist if necessary.  3. Urology Consultant : If you have a lump on your testicle, it's advisable to see a urologist for a more specialized evaluation. You can schedule an appointment with a urology consultant within the next week if it's not an emergency.  Ultimately, it's essential to take any changes or abnormalities in your testicles seriously, but the level of urgency can depend on your specific symptoms and risk factors. If you are in doubt or experiencing significant discomfort, it's always a good idea to err on the side of caution and seek medical attention promptly. Your healthcare provider can help determine the appropriate course of action based on your individual situation. |
|  |  |  |  |  |
| Haematuria (English) | There's pee in my blood but it doesn't hurt so I'm quite sure it's not UTI. there's a tight discomfort on my lower belly (abdominal area? not the stomach definitely). Here's the situation. I only realized this the first time was last Friday (15 April) night time. I went to pee and realized that my lower abdominal area had this super uncomfortable feeling like as if I was having cramps that kind of pain. And when I looked at my pee I realized that it was so red, there's definitely blood in my pee. (…) Here's even more details. I'm sexually active. My SO and I we use protection; condoms. The thing is that we've been having sex since last year and it has never happened before, only recently. The first time I had this bleeding, the day before that we had sex. I'm bleeding again and yesterday I had sex.  What could this be? Please name all the possible diagnoses! | I'm not a doctor, but I can provide some general information about the possible causes of blood in the urine (hematuria) and discomfort in the lower abdominal area. It's essential to understand that blood in the urine should always be evaluated by a healthcare professional. Possible causes of hematuria and lower abdominal discomfort may include:  1. **Urinary Tract Infection (UTI)**: While you mentioned that it doesn't hurt when urinating, UTIs can sometimes cause blood in the urine without the classic painful symptoms. Other UTI symptoms might include frequent urination, a strong urge to urinate, and cloudy or foul-smelling urine.  2. **Kidney Stones**: The presence of kidney stones in the urinary tract can lead to hematuria and lower abdominal discomfort. Kidney stones can cause intense pain if they move.  3. **Sexually Transmitted Infections (STIs)**: Certain STIs, such as chlamydia or gonorrhea, can cause urinary symptoms, including blood in the urine. It's important to get tested if you suspect an STI.  4. **Trauma or Injury**: Recent sexual activity may sometimes lead to minor trauma or irritation in the urinary or genital area, resulting in blood in the urine.  5. **Prostate Issues**: Prostate problems, such as prostatitis or an enlarged prostate, can lead to blood in the urine and lower abdominal discomfort.  6. **Bladder or Kidney Infections**: Infections of the bladder or kidneys can cause hematuria and abdominal discomfort.  7. **Medications or Blood Thinners**: Some medications or blood thinners can lead to bleeding in the urinary tract.  8. **Other Medical Conditions**: There are various other medical conditions that could potentially lead to blood in the urine, including benign or malignant (cancerous) growths.  Considering that you've observed blood in the urine on more than one occasion and experienced lower abdominal discomfort, it is essential to seek medical attention promptly. I recommend contacting a healthcare provider, preferably a urologist, to discuss your symptoms, undergo necessary tests, and receive a proper diagnosis and treatment. It's crucial to address this issue to determine the underlying cause and receive appropriate care. | Given the previous information, would you suggest presenting in the Emergency Department immediately, at the GP office tomorrow or at the office of a urology consultant within the next week? | The presence of blood in the urine (hematuria) should not be ignored, but it may not always require a visit to the Emergency Department unless you experience severe pain, other concerning symptoms, or believe it's a medical emergency. Here's a general guideline for seeking care:  1. **GP Office**: You can start by contacting your general practitioner (GP) to discuss your symptoms. They can perform an initial assessment, order tests, and provide guidance. If your GP believes your condition requires further evaluation, they may refer you to a specialist or order additional tests.  2. **Urology Consultant**: If your GP suspects a urological issue or believes you need further investigation, they may refer you to a urologist. In this case, you can make an appointment with a urology consultant within the next week.  3. **Emergency Department**: If you experience severe pain, additional concerning symptoms, or believe it's a medical emergency, such as a high fever or inability to urinate, you should go to the Emergency Department immediately.  While the presence of blood in the urine may not always be an emergency, it's crucial to get it evaluated promptly to determine the underlying cause. You should reach out to your GP as soon as possible to start the evaluation process and get the appropriate care and guidance. Your healthcare provider will assess your condition and recommend the most suitable course of action based on your specific situation. |
|  |  |  |  |  |
| Urinary tract infection (English) | Does anyone else get that mild (un comfortable not painful though) burning sensation after peeing. Like the pee is stuck half way or something and needs to get out? It's not always but it happens once in a while. It's like a mild burn, with a sensation to pee, yet I just did.What could this be? Please name all the possible diagnoses! | I'm not a doctor, but I can offer some general information about potential causes of a burning sensation during or after urination. It's important to note that if you're experiencing this symptom, you should consult a healthcare professional for a proper evaluation and diagnosis. Here are some possible diagnoses that could be associated with a burning sensation during or after urination:  Urinary Tract Infection (UTI): UTIs are common and can cause symptoms like a burning sensation, frequent urination, and a strong urge to urinate.  Sexually Transmitted Infections (STIs): Certain STIs, such as chlamydia and gonorrhea, can cause discomfort or burning during urination.  Bladder Infection (Cystitis): Infections in the bladder can lead to burning sensations and a frequent need to urinate.  Kidney Stones: If a kidney stone is blocking the urinary tract, it can cause pain and discomfort, including a burning sensation.  Interstitial Cystitis (Painful Bladder Syndrome): This chronic condition can result in a persistent burning or discomfort in the bladder area.  Urethritis: Inflammation of the urethra, which is the tube that carries urine from the bladder, can cause a burning sensation during urination.  Medications: Some medications or treatments, such as radiation therapy, can irritate the urinary tract and lead to discomfort during urination.  Dehydration: Insufficient fluid intake can result in concentrated urine, which may cause a burning sensation.  Allergic Reaction: In rare cases, an allergic reaction to certain soaps, lotions, or personal hygiene products can lead to irritation and a burning sensation.  Vulvovaginal Infections: Infections in the genital area, such as yeast infections or bacterial vaginosis, can sometimes cause discomfort that is felt during urination.  These are just some potential diagnoses, and there may be other less common causes as well. It's essential to seek medical attention if you experience a burning sensation during or after urination, as the underlying cause can vary widely, and proper diagnosis is crucial for effective treatment. A healthcare provider can perform tests, such as a urine analysis, to determine the cause of your symptoms and recommend appropriate treatment. | Given the previous information, would you suggest presenting in the Emergency Department immediately, at the GP office tomorrow or at the office of a urology consultant within the next week? | The urgency of seeking medical attention for a burning sensation during or after urination depends on several factors, including the severity of your symptoms, any additional symptoms you may be experiencing, and your medical history. Here are some general guidelines to help you decide where to seek medical care:  Emergency Department (ED): You should consider going to the ED immediately if you experience severe pain, high fever, blood in your urine, or if the burning sensation is accompanied by symptoms like vomiting or severe lower abdominal pain. These symptoms could be signs of a more serious condition that requires immediate evaluation and treatment.  GP (General Practitioner): If your symptoms are mild to moderate, not associated with severe pain or other concerning symptoms, and you are generally in good health, it may be appropriate to schedule an appointment with your GP or primary care physician for evaluation. They can assess your condition and determine if any further specialist referrals or tests are needed.  Urology Consultant: If your symptoms persist or worsen after seeing your GP, or if you have a history of recurrent urinary tract issues, it may be advisable to schedule an appointment with a urology consultant. Urologists specialize in urinary and reproductive tract disorders and can provide more specialized care and testing if necessary.  Ultimately, the decision on where to seek care should be based on your individual circumstances and the severity of your symptoms. If you are unsure about the urgency, it's generally better to err on the side of caution and seek medical attention sooner rather than later. You can also contact your GP's office for advice on whether your symptoms require immediate attention or can wait until a regular appointment.  Remember that this information is not a substitute for professional medical advice, and it's essential to consult with a healthcare provider for a proper evaluation and guidance on your specific situation. |
|  |  |  |  |  |
| Urolithiasis (English) | I have severe pain in right flank when moving, which feels like spasms and a dull ache the rest of the time.  What could this be? Please name all the possible diagnoses! | I'm not a doctor, but I can offer some general information about possible causes of severe pain in the right flank. It's important to remember that you should consult a healthcare professional for an accurate diagnosis and appropriate treatment. Some potential causes of right flank pain include:  1. **Kidney Stones**: These are hard deposits of minerals and salts that can form in the kidneys and may cause intense, spasmodic pain as they move through the urinary tract.  2. **Kidney Infection (Pyelonephritis)**: This is a bacterial infection of the kidneys that can lead to severe pain in the flank, along with other symptoms like fever and urinary issues.  3. **Muscle Strain**: Overexertion or injury to the muscles in the flank region can cause pain, and this might feel like spasms or a dull ache.  4. **Appendicitis**: While the appendix is typically located in the lower right abdomen, its inflammation can sometimes cause referred pain in the right flank area.  5. **Gallstones**: These can cause pain in the upper-right abdomen, but the pain may radiate to the right flank in some cases.  6. **Urinary Tract Infection (UTI)**: UTIs can lead to discomfort in the lower abdomen or flank area.  7. **Diverticulitis**: Inflammation of small pouches in the colon (diverticula) can cause pain in the lower abdomen that may radiate to the right flank.  8. **Inflammatory Bowel Disease (IBD)**: Conditions like Crohn's disease or ulcerative colitis can cause abdominal pain, which might be felt in the flank.  9. **Musculoskeletal Issues**: Conditions like costochondritis or rib fractures can also cause pain in the flank region.  10. **Herniated Disc**: If a herniated disc in the spine compresses nerves, it can cause referred pain to the flank area.  11. **Endometriosis**: In women, endometriosis can cause pelvic pain that may radiate to the flank.  12. **Pancreatitis**: Inflammation of the pancreas can cause pain in the upper abdomen that might be felt in the right flank.  13. **Referred Pain from Other Organs**: Pain from the liver, lungs, or other organs can sometimes be felt in the right flank.  Please keep in mind that the exact cause of your pain can only be determined by a healthcare professional after a thorough evaluation, which may include physical examination, medical history, and potentially imaging or laboratory tests. If you're experiencing severe or persistent pain in your right flank, I strongly recommend that you seek medical attention promptly. | Given the previous information, would you suggest presenting in the Emergency Department immediately, at the GP office tomorrow or at the office of a urology consultant within the next week? | I'm not a doctor, but I can offer some general guidance. The urgency of seeking medical attention for your right flank pain depends on the severity and other associated symptoms. If you are experiencing any of the following, you should consider going to the Emergency Department immediately:  1. Severe and unbearable pain that is not relieved with over-the-counter pain medications. 2. Signs of a possible kidney infection, such as a high fever, chills, nausea, or vomiting. 3. Blood in your urine. 4. Difficulty urinating or unable to urinate.  If your pain is severe, persistent, and associated with any of the above symptoms, it's important to seek immediate medical care in the emergency room.  If the pain is more moderate and you don't have the above concerning symptoms, you could consider making an appointment with your general practitioner (GP) as soon as possible. Your GP can perform an initial evaluation and determine whether further tests or specialist referrals, such as urology, are necessary.  Ultimately, the decision on where to seek medical care should be based on the severity of your symptoms and your own judgment. If you are uncertain or your pain worsens, it's always safer to seek immediate medical attention in the emergency room. Your health should be the top priority. |
|  |  |  |  |  |
| Priapism (English) | I was seeing a doctor about my depression, and he prescribed me a few meds. One of them was a medication called trazadone (used as a sleep aid). I took 50mg (a small dose) the first night, did not help with anything, and took the 2nd dose of 100mg the following night. It did help me sleep. I woke up with morning wood, which is pretty common for me. Went to pee, didn't help. Tried "the usual" to get it to go down, no luck. At this point it was not painful or anything so I just started going about my morning routine getting ready for work. I work for an engineering firm doing construction monitoring, materials testing, and on this particular day I was coring asphalt pavement to be taken to a lab for analysis. But I digress. As i am putting on my boots about an hour later I realize it wont go down. I "try again" and everything is still functional, but the after getting off "relaxation" of the penis just didn't occur. I begrudgingly get in my truck with it tucked away and go to work, praying it will relax by the time I get to the office and pick up my gear. Now I am going to skip forward in time until after work, because nothing other than being annoyed about this happened. I get back to the office, drop off my gear, and get home. For reference, I got up at 4am, and this is now about 1pm. I remembered those commercials that say if you have an erection longer than 4 hours, go see a doctor. Realizing im on hour 9, i start to panic google how i can fix this myself. I try one more round of "the usual remedy" and no dice. I ice it, and nothing. I take a cold shower, and nothing. its now 3 pm and Im super panicking.  What could this be? Please name all the possible diagnoses! | I'm not a doctor, but I can offer some general information about your situation. If you've experienced an erection that has lasted for more than four hours, it's important to seek immediate medical attention. This is a condition known as priapism, and it can be a medical emergency. Priapism can have several potential causes, and a healthcare professional will need to evaluate your specific case to determine the underlying cause and provide appropriate treatment.  Potential diagnoses for priapism include:  1. Ischemic (low-flow) priapism: This is the most common form of priapism. It occurs when blood becomes trapped in the erectile tissues, often due to a blockage of blood flow. It can be associated with conditions such as sickle cell disease, certain medications, or other medical conditions.  2. Non-ischemic (high-flow) priapism: This type is less common and typically occurs when there's an abnormal connection between an artery and a vein in the penis. It's usually not painful and is less likely to be a medical emergency.  3. Medication-induced priapism: Certain medications, including trazodone, can sometimes lead to priapism as a side effect.  4. Other potential causes: Trauma or injury to the genital area, neurological conditions, or other underlying medical issues may also lead to priapism.  The key point is that priapism should not be ignored, and seeking immediate medical attention is crucial. A healthcare professional will assess your condition, possibly drain the blood from the penis, and address the underlying cause.  In your case, it's possible that the trazodone you took may have contributed to this condition, as priapism can be a rare side effect of some medications. However, only a doctor can provide a proper diagnosis and appropriate treatment, so it's essential that you contact a healthcare professional as soon as possible to address the issue. | Given the previous information, would you suggest presenting in the Emergency Department immediately, at the GP office tomorrow or at the office of a urology consultant within the next week? | If you've been experiencing priapism (an erection lasting more than four hours), it's considered a medical emergency, and you should seek immediate medical attention by going to the Emergency Department or an urgent care facility. Priapism can lead to serious complications, including damage to the erectile tissue if not treated promptly.  It's not advisable to wait until tomorrow or schedule an appointment with a GP or urologist in the coming week, as this condition requires immediate evaluation and treatment. The emergency department is the most appropriate place to seek immediate medical assistance for priapism.  Please don't delay and go to the nearest emergency department as soon as possible to address this issue. |

Appendix Table 3: Showing one exemplary input for each of the six symptomologies into GPT-3.5. Inputs were derived from English online fora combined with the last paragraph asking for differential diagnoses. See Appendix Table 1 for sources.
